# Anterior colporrhaphy: a standard operation? Systematic review of the technical aspects of a common procedure in randomized controlled trials

**DOI:** 10.1007/s00192-017-3510-5

**Published:** 2017-12-06

**Authors:** Ksenia Halpern-Elenskaia, Wolfgang Umek, Barbara Bodner-Adler, Engelbert Hanzal

**Affiliations:** Division of General Gynecology and Gynecologic Oncology, Department of Obstetrics and Gynecology, Medical University Vienna, Waehringer Guertel 18, 1090 Vienna, Austria

**Keywords:** Colporrhaphy, Cystocele, Prolapse, Surgery, Outcome

## Abstract

**Introduction:**

Anterior colporrhaphy (AC) is considered a standard procedure and is performed all over the world. However, not a single step of the procedure has ever been truly standardized and the rates of failure show a wide range in the literature from 0% up to 92%. The aim of this systematic review was to evaluate the differences in technique and procedure worldwide.

**Methods:**

We performed a systematic literature search up to March 2016 using the MeSH terms “(anterior AND (colporrhaph* or colporhaph* or repair* or cystocel*)” using Preferred Reporting Items for Sytematic Reviews and Meta-Analyses (PRISMA). Only randomized controlled trials (RCT) were included in the systematic review. A 14-point checklist was used to assess the quality of surgery undertaken in each RCT.

**Results:**

Forty RCTs from all over the world were included in the review**.** The indication for AC was urinary incontinence and/or pelvic organ prolapse. A detailed description of colporrhaphy was not provided even in the well-conducted RCTs. The review showed differences in each step of the procedure, in perioperative care, in anesthesia and in surgeon’ experience.

**Conclusion:**

Our results highlight the problems concerning AC with the great range in postoperative outcomes. There is diversity in the anatomical structures used in the repair, in perioperative care and in the procedure itself.

## Introduction

Anterior colporrhaphy (AC) is considered a standard procedure just as are appendectomy and tonsillectomy, and is performed all over the world. In the USA, more than 200,000 operations are done annually for pelvic organ prolapse [1] and 81% of them include AC [2]. The principle of colporrhaphy is based on the plication of the vesicovaginal fascia in the midline to reinforce the natural wall between the vagina and bladder. But is there really “a standard” and do we really speak of the same procedure when we discuss this often-performed operation in prolapse surgery? For a long time AC has remained a quasistandard, although to our knowledge not a single step of the procedure has ever been truly standardized. Even in most surgical textbooks a detailed description is not given and no clear internationally relevant guidelines exist; for example, Billingham et al. point out that “Aggressiveness of the plication and the longevity of the plication are dependent on the surgeon’s preference “ [3].

In a recent comprehensive historical review, Lensen et al. [4] found that AC has been around for approximately 150 years and the recurrence rates today appear to be similar to those a century ago [4, 5]. Estimates of rates of failure show a wide range in the literature, from an unbelievable 0% up to a devastating 92% [6, 7]. This rather disappointing scatter of results for this time-honored operation considered as a “standard” has not gone unnoticed in medical research. Lensen et al. evaluated the variation in the technique of AC among members of the Dutch Urogynecologic Society [7]. Their findings demonstrated that even within a specialized small group of urogynecologists in one country, the technique of cystocele repair shows great variety. There were variations in preoperative evaluation, variations in the intraoperative steps, and divergence in postoperative follow-up programs. These findings raise the question about worldwide differences in technique and procedure in a populations of surgeons of varied clinical backgrounds.

Therefore, the aim of this systematic review was to estimate worldwide variations in the technique of AC. To our knowledge, this is the first systematic review investigating this topic.

## Materials and methods

We performed a systematic literature search using the MeSH terms “(anterior AND (colporrhaph* or colporhaph* or repair* or cystocel*)”. We searched the following databases up to the April 2017: Ovid MEDLINE (from 1946), Cochrane Central Register of Controlled Trials, and Embase (from 1974). Only randomized controlled trials (RCT) were included in the systematic review, presenting level I scientific evidence with a detailed description of the intervention [8]. Two authors (E.H., K.H.-E.) independently assessed each individual study and the steps of the procedure using Preferred Reporting Items for Sytematic Reviews and Meta-Analyses (PRISMA) [9]. The protocol of this review was registered with the PROSPERO International Prospective Register of Systematic Reviews (CRD42017065995). The following 14-point checklist was used to assess the quality of surgery in each RCT:

1. Standardization
2. Preoperative antibiotics (use, technique, duration)
3. Catheterization (use, technique, time of insertion, duration) and management of urine residual.
4. Infiltration (use, technique)
5. Incision (method, anatomical position, length)
6. Preparation of the vesicovaginal fascia (method, landmark)
7. Anatomical definition of the plicated structure
8. The plication of fascia (suture technique, suture material, description of suture length/number of sutures/distance between sutures)
9. Vaginal trimming
10. Vaginal closure (suture technique, suture material)
11. Intraoperative cystoscopy
12. Perioperative estrogen
13. Anesthesia
14. Surgeons (number, expertise)

We recorded whether steps of the procedure were standardized and which of the steps were described in detail. We also analyzed data concerning the surgeons’ qualifications, perioperative care and each step of the procedure.

## Results

**Fig. 1:**
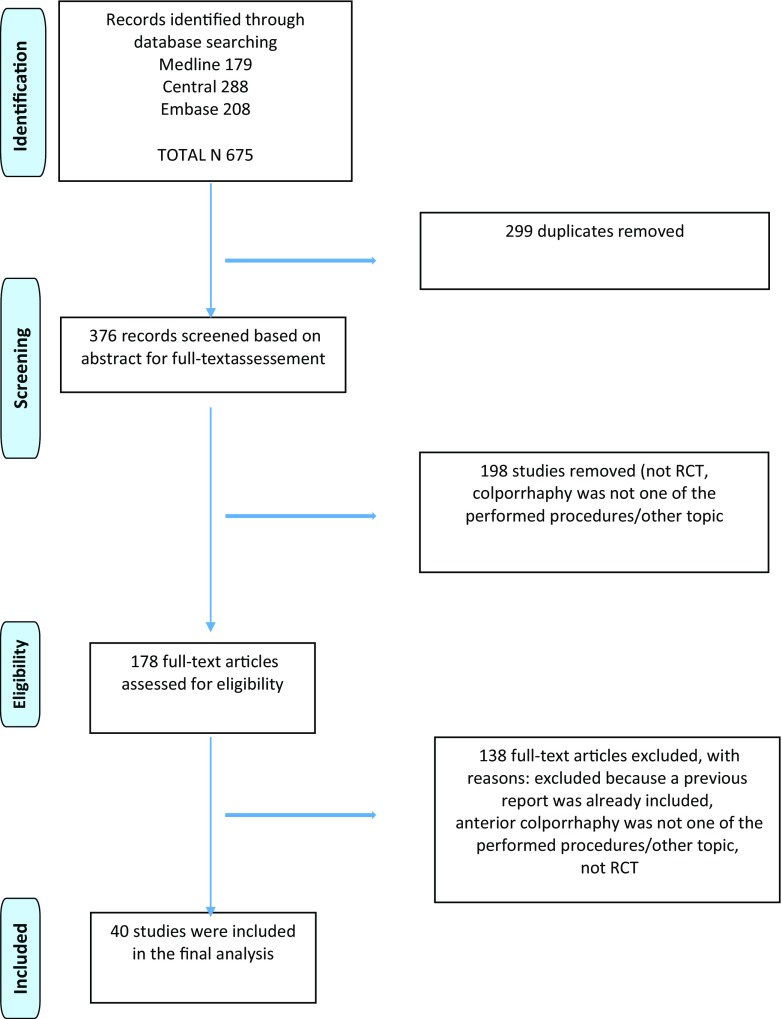
PRISMA flow chart of the study selection process

The results of the search are presented as a PRISMA flow chart (Fig. 1). Forty RCTs remained after removal of duplicates and studies not matching the eligibility criteria (Table 1). The indications for AC were urinary incontinence [10–12], pelvic organ prolapse [13, 15, 16, 19–23, 25–27, 29–38, 40, 41, 43–49] or both [14, 17, 18, 24, 28, 39, 42].

**Table 1:** Studies included for review

Reference	Country	Number of patients
[10]	USA	107
[11]	Greece	81
[12]	USA	35
[13]	USA	88
[14]	Italy	71
[15]	USA	161
[16]	USA	114
[17]	Italy	80
[18]	The Netherlands	82
[19]	USA	162
[20]	Italy	206
[21]	Finland	202
[22]	USA	76
[23]	UK	83
[24]	Greece	50
[25]	Turkey	90
[26]	USA	94
[27]	Australia	139
[28]	UK	31
[29]	Brazil	32
[30]	Denmark	31
[31]	Brazil	56
[32]	USA	55
[33]	Norway/Sweden/Finland/Denmark	389
[34]	The Netherlands	125
[35]	Sweden	135
[36]	USA	99
[37]	The Netherlands	190
[38]	Egypt	44
[39]	USA	337
[40]	Brazil	355
[41]	France	147
[42]	Iran	56
[43]	Brazil	100
[44]	Turkey	40
[45]	India	106
[46]	Norway/Sweden/Finland/Denmark	161
[47]	USA	70
[48]	Canada	57
[49]	Brazil	184

### Standardization

In nine studies (22.5%) a standardized procedure was used [10, 25, 26, 33, 35, 43, 44, 47, 48], In one study a “similar preset” [21] as defined by the authors was mentioned, but in other studies the procedure was either not standardized or was not mentioned. In none of the studies was a complete list of steps of a standardized procedure presented.

### Preoperative antibiotics

In 23 studies (57.5%) preoperative/perioperative antibiotics were administered. Data concerning antibiotic administration was not provided in the remaining studies. The duration of antibiotic therapy was provided in 17 of the 23 studies: single shot antibiotics were administered in 14 studies, with a 2-day or 3-day regimen in each [25, 45]. Information concerning the type of antibiotic was given in 11 studies (27.5%). Cephalosporins were used in four studies: first-generation (cefazolin) in two [41, 43), second-generation (cefuroxime) in one [30], and third-generation (ceftriaxone) in one [42]. Ampicillin plus clavulanic acid was used in two studies [18, 37. Combinations of two antibiotics (cephalosporin + clindamycin or metronidazole) were given in six studies: cefuroxime + clindamycin in one [33], cefuroxime + metronidazole in one [21], cefotaxime + metronidazole in one [45], cephalosporin + metronidazole in one [46], and cefazolin + metronidazole in two [37, 40].

### Catheterization and management of residual urine

In 22 studies (55.0%) no comment was provided on preoperative/postoperative catheter use. In one study catheterization was performed according to the surgeon’s preference [33]. Insertion was performed preoperatively in one study [43], postoperatively in six studies [15, 18, 19, 24, 37, 48] and intraoperatively in four studies [26, 31, 44, 45]; postoperative catheterization was not mentioned. In other studies, the time of insertion was not clear or was according to the surgeon’s preference [33]. The type of catheter used was mentioned in seven studies: in two studies a transurethral Foley catheter was used [39, 41, 44], in two a suprapubic catheter [12, 14], in one a transurethral Foley catheter placed preoperatively and replaced postoperatively with a suprapubic catheter [11], and in one either a Foley or a suprapubic catheter [15]. In seven studies the duration of catheterization was given: 24 h in three studies [40, 41, 43], “at least 2 days” in one study [14], “2–5 days” in one study [18], “72 h” in one study [32] and “5–7 days” in one study [11]. In one study a suprapubic catheter was left at least 2 days, until residual urine was less than 50 ml [14]. The management of residual urine was not mentioned in any of other studies.

### Intraoperative fluid infiltration

In 12 studies (30.0%) fluid infiltration of the operating field was performed before the incision. A vasoconstricting solution was used in two studies (vasopressin in two [19, 47], adrenaline in two [16, 38]), and the type of agent used was not given in two studies [36, 41]. A mixture of anesthetics and vasoconstricting solution was used in six studies (lidocaine and adrenaline in four [21, 26, 33, 40], adrenalin and bupivacaine in two [22, 31]). It was unclear if infiltration was performed in the remaining studies.

### Incision

Whether the colpotomy was performed with scalpel, scissors or cautery was not mentioned in any of the studies. In five studies the distance from the inferior margin of the incision to the external urethral orifice was given: 1 cm in two studies, 2 cm in two studies, and 1.5 cm in one study. In two studies the colpotomy was performed from the midurethra, in three from the urethrovesical junction, in one just below the meatus, and in one from the “proximal urethra”. In ten studies the incision was extended as far as the apex of the vagina or the cervix.

### Instruments and techniques of dissection

For preparation of the cystocele before plication the following methods were used: “sharp” (three studies), “scissors” (two studies), “pointless detachment” (one study), “blunt or sharp” (three studies), and “scissors and blunt” (one study). In 30 studies (75.0%) no information was provided on this step of AC.

### Tissue planes

Various terms were used to describe the vesicovaginal fascia and possibly other structures that were claimed to have been used for plication (Table 2).

**Table 2:** Structures used for plication

Layer	Number of studies (*N* = 40)
Fibromuscular layer	6
Pubocervical fascia	10
Endopelvic connective tissue	3
Vesicovaginal fascia	2
Pubovesicocervical fascia	1
Pubocervicovaginal fascia	1
Endopelvic fascia	1
Endopelvic fascia (fibromuscular layer)	1
Prevesical tissue	1
Underlying muscularis	1
Vesical connective tissue	1
Vesicovaginal muscularis tissue	1
Double layer: posterior wall of the bladder and paravesical fascia	1
Bladder	1
No information	9

### Anatomical landmarks

In 14 studies the anatomical limits of the preparation were mentioned and were described as follows: “median border of the decent pubic rami” (three studies), “the lateral sulci” (two studies), and (one study each) “inferior brim of the symphysis pubis”, “inferior pubic ramus”, “limits of pubic rami”, “pubic rami”, “the level of vaginal sulcus and urogenital diaphragm”, “ischio-pubic rami”, “the bladder base”, “vaginal sulci and proximally” and “white line“.

### Plication techniques and suture material

In 19 studies (47.5%) interrupted sutures were used. The characteristics of the sutures are presented in Table 3. In one third of the studies no information was provided on the type of suture material, and a diverse range of suture materials were used in the remainder. The number of stitches was given in two studies [11, 31] but no information was provided on the length of the stitches or the distance between them.

**Table 3:** Suture characteristics

Characteristic	Number of studies (*N* = 40)
Material	
Polyglactin	16
PDS	5
Chromic catgut	2
Polyethylene/polypropylene	1/1
PDS or polyglactin	1
Polyglactin + Prolene	1
Not stated	13
Absorbability	
Absorbable	27
Nonabsorbable	2
Absorbable or nonabsorbable	1
Absorbable + nonabsorbable	1
Not stated	9
Structure	
Braided	20
Monofilament	8
Braided + monofilament	1
Not stated	11
Size	
2-0	12
1-0	2
0 or 2-0	1
0 + 2-0	1
0	10
Not stated	14
Technique	
Interrupted	19
Continuous	1
Purse string	1
Not stated	19
Number of sutures	
Four to six	1
Two figures of eight	1
Not stated	38

### Vaginal trimming

In 18 studies trimming of the vagina was mentioned: trimming performed (12 studies), trimming optional/as required (three studies), and no trimming (three studies). In 22 studies no information on trimming was provided.

### Vaginal closure

In 19 studies the material used was reported: Vicryl 2-0 (16 studies), and absorbable/delayed absorbable (three studies). A continuous suture was used in 12 studies (unlocked in two, locked in six, and no information in four) and interrupted suture in five studies (figure of eight in one, and overlapping for “prevention of trimming” in one).Intraoperative cystoscopy was mentioned in four studies (10%). In two, the cystoscopy was performed according to protocol, and in two according to the surgeon’s preference [33, 40].

### Perioperative estrogen

Preoperative application of estrogen was mentioned in seven studies [10, 13, 25, 33, 41, 46, 48]. In two studies it was used in postmenopausal women for 4–6 weeks before surgery and for 3–4 weeks after surgery. In three studies the postoperative use of estrogen was recommended, but the duration of the proposed therapy was not mentioned. In one study 74% of the patients were treated with local estrogen preoperatively. In one study local estrogen at the time of study inclusion until 3 months after surgery was recommended.

### Anesthesia

Anesthesia was described in eight studies (20.0%) as follows: spinal (two studies), “in 90% spinal” (one study), regional (one study), doctor’s preference (one study), general (one study), epidural or spinal (one study), and general or regional (one study).

### Surgeons

The number of surgeons was reported in 15 studies and ranged from 1 to 22. In one study “two surgeons performed the majority” of the operations, in five studies many surgeons and/or many centers participated, but the number of surgeons was not reported. Whether surgery was performed by a qualified urogynecologist was not reported in any of the studies. In nine studies (22.5%) some information was provided concerning the surgical team with the following heterogeneous descriptions: “same surgical team”, “surgeons with trainees”, “all qualified”, “experienced surgeons”, “house staff and one of three study surgeons”, “senior residents under supervision of the two senior authors“, “surgeons, supervised by a single physician“, and “surgeons from eight hospitals, who met at two workshops“.

## Discussion

This review shows that even within the strict boundaries of RCT protocols, there is wide variation in the preoperative, procedural and postoperative steps in AC, which has been seen as a quasistandard for a very long time. A detailed description of colporrhaphy was not provided even in the well-conducted RCTs, and even the most basic anatomical structures used in the repair were unclear in many studies.

A recent Cochrane review [50] compared AC with native tissue and other techniques (including biological grafts and meshes). The review included 33 RCTs and over 3,300 procedures, and showed that all other techniques provide only minimal advantage compared with native tissue repair. Native tissue repair was associated with reduced risk of de novo stress urinary incontinence (SUI), reduced bladder injury, and reduced rates of repair surgery for prolapse, SUI and mesh exposure. Although it did not evaluate the differences in “traditional” AC, this review highlights the continuing importance of AC as the evidence is not supportive of the use of mesh or graft in preference to native tissue for repair of anterior compartment prolapse.

The 40 RCTs included in this review were from all continents (Table 1), and the review proved the hypothesis that “classical” colporrhaphy does not exist. The problem of standardization in surgery is a well-known and a widely discussed issue in the medical community. It seems plausible that standardization of the surgery all over the world is either not always possible or even meaningful. However, the growing adherence to reporting guidelines for studies, such as Consolidated Standards of Reporting Trials (CONSORT) [51] and Strengthening the Reporting of Observational Studies in Epidemiology (STROBE) [52], is beginning to shape the landscape of research of innovations in surgery and other invasive therapies. It is indeed desirable to describe interventions thoroughly, including control interventions to minimize bias attributable to the imprecision of a poorly standardized operation. The description should allow a clinician wanting to use the intervention to know exactly how to perform the intervention that was evaluated in the trial [53, 54].

The Idea, Development, Exploration, Assessment, Long-term follow-up (IDEAL) collaboration has been established to improve the quality of research in surgery, and it recommends that each procedure, including established ones, should be monitored with prospective databases [55]. These recommendations seem absolutely applicable and important for AC. We included only RCTs as they provide level one evidence for surgery. In these studies AC was used as a control intervention for many new surgical techniques for the repair of cystocele, including synthetic meshes. Often due to the failure of colporrhaphy, the studies favored the new surgical technique. However, in light of the US Food and Drug Administration (FDA) warning concerning problems with transvaginal mesh, AC using native tissue for prolapse repair still plays an important role in pelvic floor reconstructive surgery. This review demonstrated that the operation referred to as “anterior colporrhaphy” is not the same procedure worldwide, and we need a more precise description to be able to evaluate outcomes and possible reasons for failure. We not only found many differences in each step of the procedure, but also in perioperative care, anesthesia and surgeons’ experience. These are all important factors influencing the outcome of the operation.

### Conclusions

This review showed not only differences in each step of the AC procedure, but also in perioperative care, anesthesia and surgeons’ experience. These are all important factors influencing the outcome of the operation. The review highlights the problems concerning AC, which are most likely applicable to other surgical interventions. There is diversity in the anatomical structures used in the repair, in perioperative care and in the procedure itself. On the way to global communication of research results to improve patient outcomes, we should increasingly be aware that standardization of surgery is an important item on the agenda. Exact and replicable descriptions of the procedure and assessments of surgical performance should therefore be mandatory in every study of surgery and especially in future RCTs.
